# Predicting the onset of Alzheimer’s disease and related dementia using Electronic Health Records: Findings from the Cache County Study on Memory in Aging (1995–2008)

**DOI:** 10.21203/rs.3.rs-4414498/v1

**Published:** 2024-06-07

**Authors:** Karen C. Schliep, Jeffrey Thornhill, JoAnn Tschanz, Julio C. Facelli, Truls Østbye, Michelle K. Sorweid, Ken R. Smith, Michael Varner, Richard D. Boyce, Christine J. Cliatt Brown, Huong Meeks, Samir Abdelrahman

**Affiliations:** University of Utah Health; University of Utah Health; Utah State University; University of Utah Health; Duke University; University of Utah Health; University of Utah; University of Utah; University of Pittsburgh; University of Utah; University of Utah; University of Utah Health

**Keywords:** Dementia, Diagnosis, Machine learning, Medical records, Prospective cohort, Alzheimer’s disease

## Abstract

**Introduction:**

Clinical notes, biomarkers, and neuroimaging have been proven valuable in dementia prediction models. Whether commonly available structured clinical data can predict dementia is an emerging area of research. We aimed to predict Alzheimer’s disease (AD) and Alzheimer’s disease related dementias (ADRD) in a well-phenotyped, population-based cohort using a machine learning approach.

**Methods:**

Administrative healthcare data (k=163 diagnostic features), in addition to Census/vital record sociodemographic data (k = 6 features), were linked to the Cache County Study (CCS, 1995–2008).

**Results:**

Among successfully linked UPDB-CCS participants (n=4206), 522 (12.4%) had incident AD/ADRD as per the CCS “gold standard” assessments. Random Forest models, with a 1-year prediction window, achieved the best performance with an Area Under the Curve (AUC) of 0.67. Accuracy declined for dementia subtypes: AD/ADRD (AUC = 0.65); ADRD (AUC = 0.49).

**DISCUSSION:**

Commonly available structured clinical data (without labs, notes, or prescription information) demonstrate modest ability to predict AD/ADRD, corroborated by prior research.

## 1. Introduction

### 1.1 Overview

An estimated 6.7 million Americans ages 65 and older are living with dementia in 2023, with this number expected to rise to 12.7 million by 2050.^1^ Due to the lack of definitive biomarkers, effective treatments, and stigma of a diagnosis, up to 50% of Americans with dementia never receive a dementia-related diagnosis.^1-3^ Fewer yet receive a correct diagnosis, regarding etiology or underlying pathology, such as Alzheimer’s disease (AD) compared to vascular dementia, Frontotemporal Dementia, or some other related dementia (ADRD) including mixed pathology.^4^ Predictive models that can detect early signs and symptoms of dementia, or prodromal dementia, may be useful for improving care and patient outcomes.^5^ Routinely collected health data, including electronic health records (EHR) in large population cohorts, is a promising vehicle for dementia prediction. However, to date, few EHR-based prediction studies for dementia have included research cohorts with gold-standard measures for cognitive assessment.

There are over sixty studies using machine learning (ML) or deep learning (DL) to predict dementia. Approximately one-third of the models to date are based on using neuroimaging to predict transition from mild cognitive impairment to dementia while an additional third are devoted to using voice recordings to identify dementia.^6^ Of the over twenty-five studies using clinical features to predict dementia, most use features derived from cognitive screening workups including neuropsychological test scores and genetic/biomarker testing and include small sample sizes (i.e., < 500).^6^ Of the nearly dozen ML/DL dementia prediction studies using routinely collected health records, all include clinical notes, medication history, and/or lab test results in addition to diagnostic and procedural codes, and basic sociodemographic data. ^5,7-16^ We are unaware of any prior study using commonly available, structured, routinely collected health data to predict “gold-standard” based dementia diagnoses within a large population-based sample of individuals.

To address this research gap, we used numerous medical features (captured via Medicare, ambulatory, and inpatient record ICD diagnostic and procedure codes) along with key social features (captured via vital records or Census data) to predict later expert consensus-based AD/ADRD diagnoses within the Cache County Study of Memory in Aging (CCS, 1995–2008).^17^

## 2. Methods

### 2.1 Study Population

Full details on the CCS study design and methodology, including validated survey instruments used, have been published previously.^17,18^ In brief, the CCS was a 13-year prospective epidemiological study of dementia that enrolled 90% (N = 5,092) of the Cache County, Utah permanent resident population aged 65 and older as of January 1, 1995. ^17,18^ The primary purpose of the CCS was to examine genetic, psychosocial, and environmental risk factors for late-life cognitive decline. The initial interviews with CCS participants occurred in 1995, with three follow-up waves occurring 3, 7, and 10 years later. For each wave, a multi-stage dementia ascertainment protocol was employed, in which a panel of experts in neurology, geriatric psychiatry, neuropsychology, and cognitive neuroscience reviewed all available data and assigned final consensus diagnoses using National Institute of Neurological and Communicative Diseases and Stroke/Alzheimer's Disease and Related Disorders Association (NINCDS-ADRDA),^19^ Neurological Disorders and Stroke and Association Internationale pour la Recherche et l'Enseignementen Neurosciences (NINDS-AIREN) ^17^ or other standard research criteria.

Over the course of the four triennial waves of thorough dementia ascertainment among the 5,092 participants, 942 (18.5%) persons were identified with dementia (335 prevalent cases; 607 incident cases). Of these, 58% had final diagnoses (after final follow-up) of AD (probable or possible), 11% had AD comorbid with other forms of related dementia (AD Mixed), and 31% had other related dementias but no AD (ADRD). The remaining 4,150 participants (81.5%) were deemed cognitively normal or cognitively impaired no dementia (CIND) by end of follow up.^17^ Sixteen percent of participants were still alive by the end of the study in 2008. Prior work has shown high sensitivity and specificity for identifying dementia over the first two waves of ascertainment.^20,21^ The Cache County Study was approved by the Institutional Review Board. All participants gave written, informed consent to participate.

Key factors of CCS enrollees, including consensus-based dementia diagnoses (“gold standard” diagnoses), age of enrollment, race/ethnicity, and sex were linked with the 1995–2008 Master Beneficiary Summary File of the Medicare data and the 1996–2008 Utah Department of Health Hospital Facilities and Claims Records (Inpatient Hospital Claims and Ambulatory Surgery records) via the Utah Population Database (UPDB). Key additional social variables obtained from UPDB included education, rural/urban residence, occupation, and number of live births. The UPDB is a comprehensive data resource that links demographic, medical, and genealogical data for nearly all residents of Utah to support medical research.^22^ Study approvals were obtained from the Resource for Genetic and Epidemiologic Research, a special review panel authorizing access to the UPDB and the University of Utah Institutional Review Board.

### 2.2 Outcome Variables

CCS consensus-based “gold standard” dementia diagnoses were dichotomously categorized (present/absent) into the following two mutually exclusive groups: AD (AD with or without mixed pathology) and ADRD (related dementia with no AD).

### 2.3 Predictor Features

Predictor medical features used in this study were comprised of ICD diagnostic and procedure codes sourced from inpatient records, ambulatory surgery, or Medicare records. First, we removed all ICD records with an ICD-based dementia diagnosis (Supplemental Table 1). Next, we grouped remaining diagnostic codes into 65 exclusive health conditions based on Chronic Conditions Warehouse (CCW) grouping protocol (Supplemental Table 2). The 65 code groups are not comprehensive, so we added the top 99 most frequent codes found in the inpatient, ambulatory surgery and Medicare records (Supplemental Table 3) that were not included in the CCW groupings. For our baseline models we included these 164 medical features along with age of enrollment into CCS and sex assigned at birth (k = 166 features in baseline model). For our extended models, we expanded beyond age and sex to include the following five socio-demographic factors: race/ethnicity, education level, number of live births, earliest occupation, and earliest urban or rural residence captured via UPDB-linked vital and Census records (n = 171 features in extended model).

### 2.4 Prediction Window

**Figure 1:**
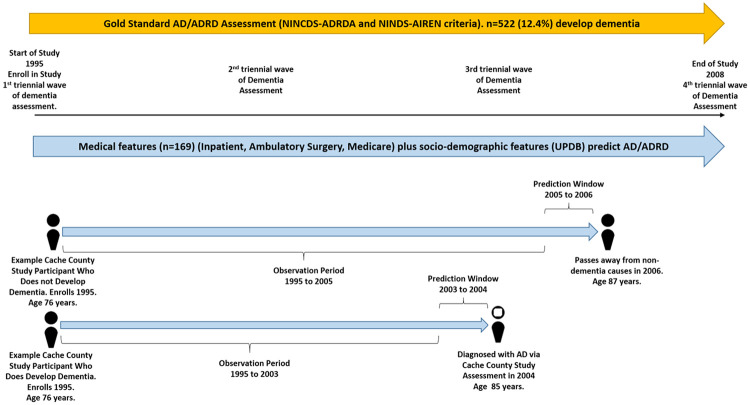
Observation period for Cache County Study of Memory in Aging participants. AD/ADRD was assessed via “gold standard” Cache County Study expert consensus assessments (four triennial waves of dementia ascertainment) according to the National Institute of Neurological and Communicative Disorders and Stroke and the Alzheimer's Disease and Related Disorders Association (NINCDS-ADRDA) and National Institute of Neurological Disorders and Stroke and Association-Internationale pour la Recherché et l’Enseignment en Neurosciences (NINDS-AIREN) criteria.

Prior research has predicted dementia based on prediction windows of anywhere from 0 to 10 years ^7,9^ meaning that the data used in these study’s prediction windows are the same number of years, or greater, prior to a participant’s dementia diagnosis date. We used the most common 1-year prediction window with up to 13 years of observation (Fig. 1).

Since our goal was to predict rather than simply classify dementia diagnoses, all predictive medical features (n = 166) that occurred after or within the same calendar year as the CCS dementia diagnosis were removed from our data. This process allowed our models to remain prospective as removal of these codes made sure the model was not trained on data that occurred after the prediction window. For CCS participants not diagnosed with dementia over the 13-year follow-up period (1995–2008), all predictive feature data were included.

**Figure 2:**
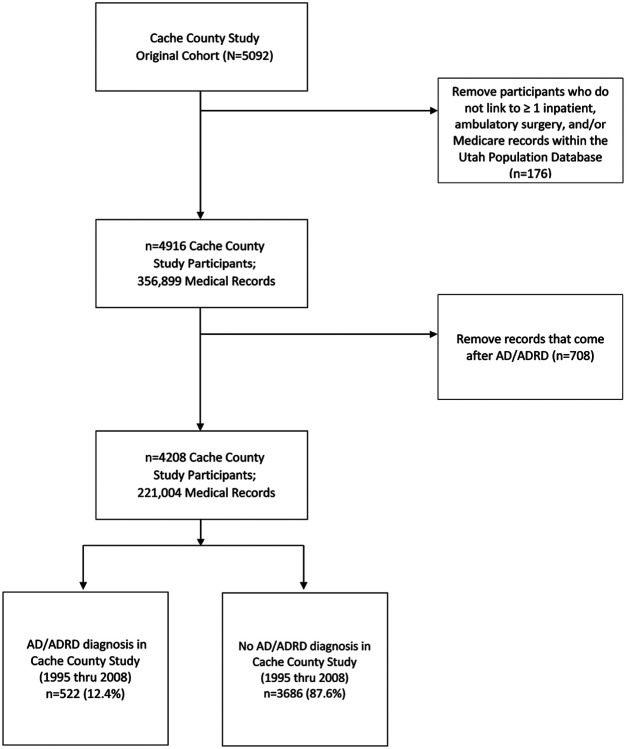
Overview of study cohort: Cache County Study in Memory in Aging participants that could be linked to the Utah Population Database (inpatient, ambulatory surgery, and/or Medicare records). Prediction modeling warranted removing records that occurred after a dementia diagnosis

After removing participants who did not link to any inpatient, ambulatory surgery or Medicare claim record (n = 176) along with removing records that came after an AD/ADRD diagnosis, 4208 (83%) of the original CCS participants, with 221,004 medical records, were included in the study (Fig. 2). Of the included participants, 522 (12.4%) had an AD/ADRD incident diagnosis over the follow-up period as per “gold standard” assessment.

### 2.5 Model Development

After data preprocessing including encoding categorical features and scaling numeric features, the data was separated into a training and testing dataset using a stratified 80/20 split for each outcome variable.^23^ Four different models were trained (Gradient Boosting Classifier [GBC], Random Forest [RF], Multi-layer perceptron [MLP], and XGBoost [XGB]) using the 166 features (171 features in extended models) for the three different outcome metrics (All-cause dementia, AD/ADRD, and ADRD). Each of the four models (GBC, RF, MLP, and XBG) were trained on the training dataset using a nested five k-fold cross validation with an inner validation of three k-folds. The trained model was tested on 10 bootstrapped samples to determine its generalizability. Python 3 was used for all analyses, with scikit-learn package used in conjunction with the imblearn package for implementing imbalance techniques.

### 2.6 Sensitivity Analysis I: Expanding to include ICD-based dementia diagnoses

Given the high specificity of dementia diagnoses within administrative healthcare records,^3,4^ we additionally ran models where we included the CCS “gold-standard” dementia diagnoses and the ICD-based diagnoses. Furthermore, given repeated clinic encounters in our ICD-based administrative healthcare database, we estimated models where we required participants to have one ICD dementia diagnosis to be considered a dementia case and where we required at least three ICD dementia diagnoses to be considered a case.^9,24^

### 2.7 Sensitivity Analysis II: Not eliminating records that came after dementia diagnosis (Classification modeling)

Given that dementia researchers using administrative healthcare records for retrospective cohort analyses are interested in correctly classifying dementia, we additionally ran models whereby we did not exclude medical features coming after the dementia diagnosis.

### 2.8 Sensitivity Analysis III: Addressing potential selection bias by including all CCS participants, regardless of whether they had medical record linkage or not.

Given potential selection bias by excluding individuals who did not link to ≥1 inpatient, ambulatory surgery, and/or Medicare records within the UPDB but who did have sociodemographic features available (n = 5091), we ran our models by including all participants, regardless of whether they had medical record linkage.

## 3. RESULTS

### 3.1 Study Population Characteristics

The average age of enrollment among included CCS participants was 76.3 ± 7.0 years. The study comprised 56% females, 98% white non-Hispanics, 84% with less than a college education, and average number of live births of 2.1 ± 2.6. Farming and homemaking were the most common occupations listed, making up 29% of the total occupations. The majority were considered to live in urban areas (88%).

### 3.2 Prediction models using combined datasets

Gradient Boosting Trees (GBC) and Random Forest (RF) achieved the best performances for predicting dementia on the test set (AUC: 0.65 and 0.67, respectively, for all-cause dementia) (Table 1). AUCs declined when evaluating dementia subtypes: AD/ADRD: GBC AUC = 0.58 and RF AUC = 0.65; and ADRD, GBC AUC = 0.55 and RF AUC = 0.49. Results slightly improved for the RF models when adding sociodemographic features including number of prior live births, race/ethnicity, education, earliest occupation, and earliest residence for all-cause dementia AUC = 0.69 (Supplemental Table 3). Implementing techniques to address dementia imbalance in our dataset did not improve accuracy.

### 3.2 Feature Importance

**Figure 3:**
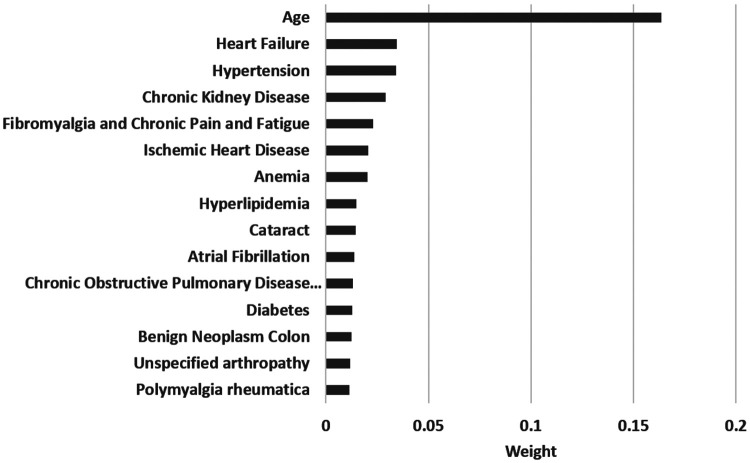
Top 15 features of importance extracted from the fit model

The most important features were extracted from the fit model and are shown in Fig. 3 for all-cause dementia. The five most influential features were age with a weight of 0.1636 followed by heart failure (0.0346), hypertension (0.0343), chronic kidney disease (0.0291) and fibromyalgia (0.0231).

### 3.3 Sensitivity Analyses

Defining dementia by using the UPDB administrative healthcare ICD records (via Medicare claims, ambulatory surgery, and inpatient records) resulted in a marked improvement in accuracy, especially when we defined dementia by having ≥ 1 ICD-based dementia diagnosis over study period (1995–2008) with an AUC = 0.78 for all-cause dementia in the GBC model and AUC = 0.77 in the RF model (Table 2). Requiring ≥3 vs ≥ 1 ICD-based diagnoses to define dementia did not result in model improvement.

There was no appreciable improvement in accuracy of classifying versus predicting dementia when we included all the medical features over the observation period, regardless of whether they came before or after a dementia diagnosis: AUC = 0.67 for GBC and AUC 0.61 for RF for all-cause dementia (Supplementary Table 4). Our models addressing selection bias where we included all 5091 participants that linked to the UPDB regardless of whether they linked to any medical records resulted in only a slight improvement in accuracy: AUC = 0.69 for GBC model and AUC = 0.67 for RF model for all-cause dementia. (Supplementary Table 5).

## 4. DISCUSSION

### 4.1 Main Findings

In this study, we evaluated commonly available health records and their ability to predict whether a CCS participant had Alzheimer’s or another related dementia disease using gold standard consensus-based diagnoses (1995–2008). Using linked datasets of inpatient, ambulatory surgery, and Medicare data, we sought to determine if 65 CCW conditions, 7 sociodemographic features and an additional 99 data-driven ICD codes would be able to train a machine learning model. We obtained only modest results with Random Forest models achieving an AUC of 0.69, 0.61, 0.50 for all-cause dementia, AD/ADRD, and ADRD, respectively in the full model of 171 features. Our models improved when using ICD-based dementia diagnoses, which makes sense given that the medical features are ICD-based diagnoses. Uncertainty regarding whether ICD-based dementia diagnoses represent true dementia warrants caution in using only ICD-based diagnoses for prediction modeling.

### 4.2 Comparison with Previous Studies

A recent systematic review reported accuracies between 64% and 99% (mean 86%) in 25 clinical studies using machine or deep learning to predict dementia.^6^ However, the majority of these studies, as well as others not listed in this review,^7 8^ included neuropsychological test scores, genetic, gait, or other blood-based biomarkers that are not part of commonly available health records in community-based samples.

There are nearly a dozen prior studies strictly using EHR and/or other routinely collected health records (e.g., Medicare claims) in their supervised machine learning dementia prediction models. ^5,7-16^ All of these studies use a combination of structured data to assemble their predictive features including sociodemographic data, ICD diagnostic and procedural codes, vital signs, medications, and lab test results; with a handful also including clinical notes.^10,12,14^ Sample sizes range from 4000 to over 5 million with prediction windows (time prior to dementia detection) ranging from 0 to 8 years and accuracies between 65% and 94%.

The vast majority of prior EHR AD/ADRD prediction studies used ICD diagnoses (sometimes accompanied by dementia medications) to define their dementia outcome. Only one study was similar to ours in using a community-based sample of individuals participating in an aging cohort study for which gold-standard dementia ascertainment was assessed as the outcome.^5^ Barnes et al conducted a retrospective cohort study among 4330 participants in the Adult Changes in Thought (ACT) study who underwent a comprehensive dementia assessment every two years and have linked EHR data including socio-demographics (age, sex, and race/ethnicity), 31 medical conditions via ICD-9 codes, vital signs (body mass index and blood pressure), healthcare utilization and medications for a total of 64 predictors. Splitting the data into a 70:30 training and test set, they applied the LASSO approach to predict unidentified dementia (i.e., dementia identified via ACT assessments but not reported in the EHR) using the EHR information for the prior two years arriving at AUCs of 0.81 (95% CI 0.78, 0.84). While this study is similar to ours in using a community-based sample and gold-standard assessment for dementia diagnoses, comparison between this study and ours is difficult since we did not remove recognized dementia cases. However, authors reported that using all dementia cases (recognized and unrecognized) did not improve performance.^5^ What this study does reveal is that relatively good prediction (> 80% accuracy) can be achieved in a sample of less than 5000 using only 64 knowledge-driven predictors.

The other strictly EHR AD/ADRD structured machine learning studies all used the ICD diagnostic codes to identify dementia. ^9-12,15,16^ Jammeh et al conducted a study in the UK (2010–2012) among over 26,000 eligible primary care patients (850 with dementia randomly matched to 2213 controls) and used over 15,000 diagnostic, process of care, and medication codes over a 2-year period, arriving at AUCs of 0.87 for the Naïve Bayes classification results.^16^ Li et al conducted a study using EHR records from the OneFlorida + Research Consortium of 23,835 ADRD patients randomly matched 1:10 to controls and used over 2500 sociodemographic, PheWas, RxNorm, CCS, vital signs, and lab value tests in their prediction models. The Gradient Boosting Tree models achieved the best performance with AUCs of 0.94, 0.91, 0.88, and 0.85 for prediction of ADRD 0, 1, 3, or 5 years, respectively, before diagnosis. A South Korean study (2002–2013) among 40,736 patients using 4894 features captured from the National Health Insurance Service database also found relatively high AUCs using models: AUCs of 0.90 for 0-year and 0.78 for 1-year model predicting definite AD.^11^ A retrospective analysis of 7587 patients from New York (2007–2019) who had at least 5 years of records (702 with probable AD) found slightly lower AUCs using XGBoost predictive models: AUCs of 0.76 for 0-year and 0.75 for 1-year model predicting probable AD.^15^ A study conducted among US Veterans with (n = 1861) and without (n = 9305) dementia used 853 EHR features including clinical notes to arrive at an AUC of 0.91 for dementia using logistic regression models.^12^ As we saw in our sensitivity analyses, using ICD diagnostic codes to identify dementia (rather than research, consensus-based diagnoses such as what we used from the CCS) can result in higher accuracy, but the question is whether these models are predicting true dementia. Further research using both “gold standard” and “ICD-based” diagnoses as well as validation work for dementia ascertainment in medical records is warranted.

In addition to the Barnes et al study using gold-standard assessments to capture dementia, the most relevant prior study would be that conducted by Miled et al.^10^ This study, for which dates of study were not provided, used classification to train EHR data, captured 10 years prior to index date, from the Indiana Network for Patient Care and Research through the Regenstrief Institute. The study was conducted on 2159 patients with dementia and 11,558 controls and extracted prescriptions, diagnoses, and medical notes from the EHR record of each patient. One major strength of the analyses is that the authors divided the accuracy results for the 1-year and 3-year prediction models by the different datasets used: prescription, diagnoses, and medical notes. Accuracy for the 1-year models for medications, diagnoses, and medical notes was 0.70, 0.65, and 0.74; and for the 3-year was 0.66, 0.63, and 0.70. Combining all three resulted in an AUC of 0.77 for the 1-year and 0.34 for the 3-year. While comparisons between Miled et al and our study are limited given their use of ICD codes to define dementia and having a sample of over 13,000 compared to our sample of over 4000, our results for just using EHR diagnoses is comparable with their finding an AUC of 0.65 compared to our AUC of 0.67. Clearly, running structured ML models using only EHR ICD diagnoses and procedures results in models of relatively poor performance.

### 4.3 Strengths

One of the strengths of this study is having a 13-year follow-up on a community-based sample of over 4200 well-phenotyped individuals participating in the CCS with linkages to over 220,000 medical records. To our knowledge, the Barnes et al study is the only other study that aimed to build predictive models using a gold standard research cohort linked to administrative healthcare records,^5^ whereas other studies use some form of ICD based dementia diagnosis as the main outcome variable. By using gold standard dementia diagnoses to define our outcome, we strengthen our model’s validity by ensuring that the study participants who are positive for the disease have the disease, and those study participants who are classified as not having dementia are also classified to a higher degree of accuracy. We are the first US study to use accessible, standardized Medicare claims and Department of Health facilities data (inpatient and ambulatory surgery records). While our prediction using these standardized records was modest at best, other studies using health insurance service databases in other countries augmented by medication and/or laboratory test results performed relatively well (AUCs over 0.78) meaning that these US health databases may also be informative in dementia prediction as long as medication and vital signs/lab results are included. Finally, we are the first study to assess whether our prediction models differed between Alzheimer’s disease or Alzheimer’s disease comorbid with another dementia (AD/ADRD) as compared to having a related dementia with no Alzheimer’s disease pathology (ADRD). Our finding of our models better able to predict AD/ADRD (AUC = 0.65) compared to ADRD (AUC = 0.49) need to be explored in future studies.

### 4.4 Weaknesses

Given that our source data was focused on a population over the age of 65 years and were predominately non-Hispanic white, generalizability of our findings to younger or other races/ethnicities is limited. Indeed, we found little improvement in our models when adding in our sociodemographic features, perhaps due to the limited diversity within the sample. However, Barnes et al, only found age and sex, not race/ethnicity, to be predictive of dementia in their models.^5^ Finally, we only considered dementia cases as compared to non-dementia cases. Future work whereby non-dementia cases are split between cognitively normal/no dementia versus CIND may help with model accuracy.

### 4.5 Conclusion

Overall, our machine learning models had only modest ability to predict dementia within the CCS. This is due in part to our relatively small sample of just over 4200 participants and more importantly due to strict reliance on diagnoses and procedures with limited information on sociodemographic factors. Leveraging larger dataset with more features beyond diagnosis and procedure codes, including medications, vital signs, lab test results, and clinical notes may enrich the feature set to improve each model’s training and performance in predicting whether an individual develops dementia. Furthermore, when resources allow, adding in imaging, serum, and cerebrospinal biomarkers will help in determining dementia subtypes. This in the end will lead to improved early diagnosis, care management, and ultimately better patient outcomes among at-risk individuals.

## Figures and Tables

**Table 1:** Comparison of the model performances for predicting “gold standard” All-cause dementia, AD/ADRD, or ADRD using 164 UPDB administrative healthcare record medical features, enrollment age, and sex in the Cache County Study of Memory in Aging

Model	Metric	All-cause dementia	AD/ADRD	ADRD
GBC	AUC (train)	0.6731	0.7033	0.6254
	AUC (test)	0.6520	0.5775	0.5458
	Specificity (train)	0.6917	0.8078	0.6626
	Specificity (test)	0.8290	0.8422	0.9628
	Sensitivity (train)	0.5671	0.4828	0.4817
	Sensitivity (test)	0.4751	0.3129	0.1288
	f1 (train)	0.5447	0.5673	0.4180
	f1 (test)	0.6119	0.5444	0.5477
MLP	AUC (train)	0.6660	0.6551	0.6292
	AUC (test)	0.5407	0.5572	0.5171
	Specificity (train)	0.7429	0.6996	0.7412
	Specificity (test)	0.8089	0.9141	0.9757
	Sensitivity (train)	0.4905	0.4409	0.3168
	Sensitivity (test)	0.2725	0.2003	0.0585
	f1 (train)	0.5577	0.4651	0.4284
	f1 (test)	0.5261	0.5533	0.5200
RF	AUC (train)	0.7063	0.6974	0.6301
	AUC (test)	0.6745	0.6499	0.4869
	Specificity (train)	0.7221	0.8601	0.6349
	Specificity (test)	0.7982	0.8965	0.8420
	Sensitivity (train)	0.5690	0.3778	0.5465
	Sensitivity (test)	0.5508	0.4034	0.1318
	f1 (train)	0.5667	0.5718	0.4261
	f1 (test)	0.6129	0.6175	0.4765
XGB	AUC (train)	0.5088	0.5136	0.5041
	AUC (test)	0.5000	0.5090	0.4915
	Specificity (train)	0.0000	0.1919	0.1960
	Specificity (test)	0.0000	0.9808	0.9830
	Sensitivity (train)	1.0000	0.8250	0.8143
	Sensitivity (test)	1.0000	0.0371	0.0000
	f1 (train)	0.1103	0.1689	0.1401
	f1 (test)	0.1099	0.5035	0.4840

AUC: Area Under the Curve GBC: Gradient Boosting Classifier; MLP: Multi-Layer Perception Model; RF: Random Forest; XGB: Extreme Gradient Boosting;

All-cause dementia includes individuals diagnosed with any dementia (see Supplemental Table 1). AD/ADRD: Includes individuals diagnosed only with Alzheimer’s disease or diagnosed with Alzheimer’s disease and some other Alzheimer’s Disease Related Dementia; ADRD: Includes individuals with no Alzheimer’s disease diagnosis, but some other Alzheimer’s Disease Related Dementia

**Table 2:** Comparison of the model performances for classifying ICD-based All-cause dementia, AD/ADRD, and ADRD using 166 UPDB administrative healthcare record medical features plus enrollment age and sex in the Cache County Study of Memory in Aging. Classification analyses used all medical features over the study period (1995 to 2008) for both participants who did and did not develop dementia (i.e., features that occurred after a dementia diagnosis were not removed). We provide results when dementia was defined as A) ≥ 1 ICD-based dementia diagnosis and B) ≥ 3 ICD-based dementia diagnosis

A. Dementia defined as having ≥ 1 ICD-based dementia diagnosis				
Model	Metric	ICD All-cause dementia	ICD AD/ADRD	ICD ADRD
GBC	AUC (train)	0.8595	0.7108	0.8154
	AUC (test)	0.7835	0.6132	0.7683
	Specificity (train)	0.7531	0.8582	0.7656
	Specificity (test)	0.8056	0.9270	0.8520
	Sensitivity (train)	0.8121	0.4427	0.7298
	Sensitivity (test)	0.7613	0.2995	0.6846
	f1 (train)	0.7760	0.6176	0.7299
	f1 (test)	0.7825	0.6193	0.7673
MLP	AUC (train)	0.8351	0.6119	0.7403
	AUC (test)	0.7327	0.6016	0.6965
	Specificity (train)	0.7489	0.8291	0.7655
	Specificity (test)	0.7431	0.8670	0.8470
	Sensitivity (train)	0.7977	0.3461	0.6384
	Sensitivity (test)	0.7223	0.3363	0.5461
	f1 (train)	0.7674	0.5610	0.6928
	f1 (test)	0.7303	0.5848	0.7024
RF	AUC (train)	0.8582	0.7132	0.8199
	AUC (test)	0.7686	0.6335	0.7643
	Specificity (train)	0.7474	0.7960	0.7760
	Specificity (test)	0.8194	0.8787	0.8202
	Sensitivity (train)	0.7999	0.4926	0.7208
	Sensitivity (test)	0.7178	0.3882	0.7084
	f1 (train)	0.7676	0.5846	0.7336
	f1 (test)	0.7698	0.6143	0.7564
XGB	AUC (train)	0.7408	0.5387	0.6798
	AUC (test)	0.7334	0.5258	0.6916
	Specificity (train)	0.8126	0.1906	0.8358
	Specificity (test)	0.8034	0.9499	0.8497
	Sensitivity (train)	0.6691	0.8341	0.5239
	Sensitivity (test)	0.6634	0.1017	0.5335
	f1 (train)	0.7427	0.1968	0.6849
	f1 (test)	0.7351	0.5283	0.6982
B. Dementia defined as having ≥ 3 ICD-based dementia diagnosis				
Model	Metric	ICD All-cause dementia	ICD AD/ADRD	ICD ADRD
GBC	AUC (train)	0.8288	0.6915	0.8159
	AUC (test)	0.7462	0.6266	0.7683
	Specificity (train)	0.8166	0.8346	0.7904
	Specificity (test)	0.8339	0.9165	0.8520
	Sensitivity (train)	0.7042	0.4383	0.7040
	Sensitivity (test)	0.6585	0.3367	0.6846
	f1 (train)	0.7302	0.6000	0.7357
	f1 (test)	0.7272	0.6267	0.7673
MLP	AUC (train)	0.7647	0.6446	0.7941
	AUC (test)	0.6872	0.5564	0.6969
	Specificity (train)	0.8075	0.8652	0.7582
	Specificity (test)	0.8439	0.8831	0.8477
	Sensitivity (train)	0.6079	0.3368	0.7074
	Sensitivity (test)	0.5305	0.2297	0.5461
	f1 (train)	0.6865	0.5834	0.7174
	f1 (test)	0.6832	0.5521	0.7028
RF	AUC (train)	0.8277	0.7190	0.8187
	AUC (test)	0.7121	0.6179	0.7684
	Specificity (train)	0.8037	0.8424	0.8003
	Specificity (test)	0.9285	0.9236	0.8435
	Sensitivity (train)	0.6912	0.4325	0.6913
	Sensitivity (test)	0.4956	0.3122	0.6934
	f1 (train)	0.7155	0.6043	0.7368
	f1 (test)	0.7321	0.6217	0.7653
XGB	AUC (train)	0.6695	0.5647	0.6786
	AUC (test)	0.6916	0.5258	0.6916
	Specificity (train)	0.8934	0.7545	0.8406
	Specificity (test)	0.8987	0.9499	0.8497
	Sensitivity (train)	0.4457	0.3585	0.5166
	Sensitivity (test)	0.4845	0.1017	0.5335
	f1 (train)	0.6799	0.4828	0.6842
	f1 (test)	0.7030	0.5283	0.6982

AUC: Area Under the Curve GBC: Gradient Boosting Classifier; MLP: Multi-Layer Perception Model; RF: Random Forest; XGB: Extreme Gradient Boosting;

ICD: International Classification of Diseases, dementia diagnoses were obtained via the UPDB administrative healthcare records.

All-cause dementia includes individuals diagnosed with any dementia (see Supplemental Table 1). AD/ADRD: Includes individuals diagnosed only with Alzheimer’s disease or diagnosed with Alzheimer’s disease and some other Alzheimer’s Disease Related Dementia; ADRD: Includes individuals with no Alzheimer’s disease diagnosis, but some other Alzheimer’s Disease Related Dementia

## Abbreviations

AD: Alzheimer’s Disease
ADRD: Alzheimer’s Disease Related Dementias
AUC: Area under the curve
CCS: Cache County Study of Memory and Aging
CCW: Chronic Conditions Data Warehouse
DL: Deep Learning
EHR: Electronic Health Record
GBC: Gradient Boosting Classifier
ICD: International Classification of Diseases
MLP: Multi-layer perceptron
RF: Random Forest
UPDB: Utah Population Database
XGB: XGBoost
